# Beating the odds: programming proliferation in the mammalian heart

**DOI:** 10.1186/s13073-018-0550-5

**Published:** 2018-05-18

**Authors:** Rajan Jain, Andrey Poleshko, Jonathan A. Epstein

**Affiliations:** 1 Department of Medicine, Cardiovascular Institute, Perelman School of Medicine at the University of Pennsylvania, Philadelphia, PA 19104 USA; 2 Department of Cell and Developmental Biology, Cardiovascular Institute, Perelman School of Medicine at the University of Pennsylvania, Philadelphia, PA 19104 USA

**Keywords:** Regeneration, Cyclin, Myocyte, Proliferation

## Abstract

The heart is one of the least regenerative organs in the human body; adult cardiac myocytes divide at extremely low frequency. Therefore, meaningful induction of cardiac regeneration requires in-depth understanding of myocyte cell-cycle control. Recent insights into how myocytes can be coaxed into duplicating in vivo might inform emerging therapeutics.

## Regenerative capacity of the mammalian heart

Many tissues in the human body will reactivate proliferative pathways to regenerate cells upon injury. However, the heart is among the least regenerative organs in the human body, and adult cardiac myocytes rarely complete cell-cycle divisions. The limited potential of the heart to regenerate has been recognized by physicians and scientists for decades. As the prevalence of congestive heart failure grows, there is hope that a regenerative approach may be useful for a disease in which very few therapies directly target the failing cell type, the cardiac myocyte. Recent quantitative studies that used carbon dating in humans and nitrogen isotope labeling in mice have demonstrated that approximately 1% of cardiac myocytes are regenerated per year, and this number decreases with age [1, 2]. There is scant evidence of a resident or circulating cardiac progenitor cell that is capable of differentiating into mature cardiac muscle [3], despite numerous claims that have engendered much controversy. Hence, there has been sustained interest in the discovery of methods to augment the ability of existing myocytes to divide in order to regenerate functional heart muscle in settings of injury and cardiac dysfunction.

Myocytes in many lower organisms, such as the newt and zebrafish, can re-enter the cell cycle and proliferate in response to injury. However, this ability was lost during evolution of higher organisms. The inability of myocytes to proliferate is due to terminal differentiation, an irreversible commitment to the differentiated phenotype that results in a quiescent state [4]. Earlier studies in amphibian adult heart have shown that cardiac myocytes proliferate in response to injury, and cardiac myocyte proliferation is enhanced by addition of specific growth factors [5]. Recent experiments demonstrate that murine cardiac myocytes are also capable of dividing to heal myocardial injury within the first 7 days of birth. After this initial period of replicative competence, myocytes are post-mitotic and divide infrequently if at all [6]. Consistent with this loss of replicative ability, embryonic cardiac myocytes have high levels of cyclin-related gene expression and mature cardiac myocytes have nearly undetectable levels. Recent studies have provided important insights into how myocytes can be coaxed into duplicating in vivo*,* which may inform emerging regenerative therapeutics. We discuss the historical context and implications of these exciting studies.

## Regulation of the cell cycle in the mammalian heart

Improving our molecular understanding of cell-cycle control in neonatal and adult mammalian cardiac myocytes has long been the focus of study to provide the foundation for inducing regeneration of the adult heart. Gene expression studies and proteomic analyses have provided detailed information on cell-cycle checkpoint control and regulation [6, 7]. Cyclins and cyclin-dependent kinases (CDKs) have been known to tightly regulate cell-cycle progression, providing substrate specificity and kinase activity, respectively. The CDK4/Cyclin D complex regulates G1 progression and the G1/S transition, which is followed by CDK2/Cyclin A activity and initiation of DNA replication. After DNA replication, the cell progresses into G2, which is accompanied by activation of the CDK1/Cyclin B complex. Aurora kinases are activated during G2/M and regulate the M phase of the cell cycle. Differentiated cardiac myocytes exit the cell cycle at G0 [8]. Therefore, in order to re-enter the cell cycle and duplicate, a cascade of CDKs/cyclins must be re-activated to progress through the G1/S and G2/M checkpoints.

Over the past 20 years, multiple groups have attempted to coax adult myocytes to re-enter the cell cycle by using transgenic and viral infection techniques to overexpress various factors, including cyclins and regulators of ‘pocket proteins’ (such as phosphorylated retinoblastoma protein) which restrain G1/S transition. Although there have been some encouraging results, the overall regenerative response was generally suboptimal due to a failure to complete cell division, a loss of mature myocyte gene expression, or cell death. For example, exogenous expression of genes that encode adenoviral Early region 1A or transcription factor E2F-1 can bypass the G1/S checkpoint and promote DNA synthesis, but cytokinesis remains blocked at the G2/M checkpoint and the result is multi-nucleation or cell death (reviewed in [7]). Transgenic overexpression of Cyclin D1, D2, or D3 (*CCND1–3*) in cardiac myocytes (under the control of a myocardial-specific promoter) results in an increase in DNA synthesis without substantial mitosis of adult myocytes, and only *CCND2* overexpression results in increased DNA synthesis upon infusion of isoproterenol or coronary artery ligation. Encouragingly, transgenic overexpression of Cyclin A2 (*CCNA2*) in murine cardiac myocytes results in an increase in the number of proliferating cell nuclear antigen-positive or phospho-histone H3+ cells in the heart, and adenoviral delivery of Cyclin A2 after myocardial infarction in pigs produces increased myocyte mitoses and improved function (reviewed in [6]).

## Unlocking the regenerative potential of the mammalian heart

Recently, Mohamed et al. [9] leveraged these observations and others to describe an effective strategy for bypassing both G1/S and G2/M checkpoints by delivering a cocktail of genes to adult myocytes in vivo that promote proliferation.

This group defined differentially expressed genes relevant to the cell cycle in embryonic day 10.5 murine myocytes compared to neonatal and adult myocytes. Several candidates increased mitosis in cultured adult murine, rat, and human cardiac myocytes. Expression of a trio of factors, CDK1, Cyclin B1 (*CCNB*), and Aurora kinase B, increased the percentage of phospho-histone H3+ cardiac myocytes, but also led to cell death, as observed in earlier studies. The authors surmised that this might be due to the induction of a heightened DNA damage response. To bypass this effect, they screened genes that would promote stable proliferation, and discovered that a combination of four cell-cycle regulators from G1/S and G2/M—CDK1, CCNB, CDK4, and CCND2—enhance cardiac myocyte proliferation without a substantial DNA-damage response.

The clonal expansion of treated cells was rigorously assessed in vivo using myocyte-specific Cre drivers in combination with the mosaic analysis with double markers (MADM) system in mice. MADM is a mouse genetic method to fluorescently label and fate-map individual cells and their clonal derivatives. It relies upon the recombination of genes that encode fluorescent markers during DNA synthesis, which results in cells displaying one of four possible color indicators: green, red, yellow (red and green), or colorless. Mohamed et al. [9] recognized that green or red cells must represent newly formed myocytes, whereas yellow cells could represent myocytes undergoing DNA synthesis without cytokinesis. Adenovirus encoding each of the four cell cycle regulators CDK1, CCNB, CDK4, and CCND2 was introduced via intramyocardial injection, and a striking increase in the numbers of newly formed myocytes was observed. Of note, the MADM system is inefficient; therefore, the observed events are likely an underestimate of the actual events. The number of myocytes that received some or all of the viruses in these experiments is unknown, as is the stoichiometry of factors expressed by infected cells.

Introduction of the four cell cycle regulators shortly after myocardial infarction enhanced myocyte proliferation. Functional outcomes were also improved in the treatment group, though it is difficult to discern what portion of the benefit observed was due to myocyte replication versus other effects of the treatment such as the release of paracrine factors. Finally, the authors identified small molecule inhibitors of Wee1 (a negative regulator of CDK1) and TGF-β signaling that could replace CDK1 and CCNB in a modified cocktail, an important advance that partially substitutes viral factor overexpression with small molecules. Interestingly, overexpression of *CCND2* in human induced pluripotent stem cell (IPSC)-derived cardiac myocytes also increases proliferation [10], suggesting that therapeutic strategies to enhance proliferation may include either treatment of exogenous (embryonic stem cell- or iPSC-derived) cardiac cells prior to cell delivery, or treatment of endogenous cells in situ*.*

Intriguingly, another recent study indicates that exercise might be a powerful tool to induce myocyte duplication [11]. Vujic and colleagues [11] labeled newly forming DNA in adult mice by feeding them ^15^N-thymidine. Using an extremely innovative approach, they were able to visualize cells that incorporated the labeled thymidine. Remarkably, a greater than fourfold increase in the number of newly formed diploid myocytes was recorded after 8 weeks of exercise. Exercise also increased the number of newly formed myocytes after myocardial infarction, and the group demonstrated that miR-222 regulates myocyte duplication. Though the group previously demonstrated that new cardiac myocytes form from pre-existing myocytes [2], a lineage tracing approach would need to be used to confirm that the new myocytes identified after exercise originate from pre-existing myocytes. This innovative approach could be used in humans, and it would be fascinating to determine if elite athletes have higher levels of myocyte duplication or whether cardiac rehabilitation after myocardial infarction promotes cardiac regeneration.

## Conclusion and future prospects

The inability of the adult mammalian heart to regenerate adequately after injury remains an impediment to recovery after myocardial infarction. Ongoing research has identified many factors that impact cardiac regenerative capacity. Nevertheless, it is now clear that adult cardiac myocytes can be directed to re-enter the cell cycle and successfully complete cytokinesis to produce new myocytes. These studies will inform therapeutic approaches, including the administration of iPSC-derived cardiac cells, implantation of pre-formed tissues, or manipulating the ability of endogenous cardiac myocytes to proliferate. Any approach must be tailored to ensure protection from arrhythmias and the generation of a minimal host immune response. Furthermore, identification of the optimal window in which to administer each approach is important, which may be slightly different for each approach.

Further research will be needed to understand whether all cardiac myocytes, or only a specific subset, can be induced to proliferate. Advances in gene delivery to the heart, and in the basic understanding of cell-cycle regulatory control, open the path to developing effective therapies that generate new functional heart tissue from myocytes previously thought to be terminally differentiated.

## Abbreviations

CCNB: Cyclin B1
CCND: Cyclin D
CDK: Cyclin-dependent kinases
MADM: Mosaic analysis with double markers
